# Rickettsialpox in Turkey

**DOI:** 10.3201/eid0911.030224

**Published:** 2003-11

**Authors:** Mustafa K. Ozturk, Tamer Gunes, Mehmet Kose, Christopher Coker, Suzana Radulovic

**Affiliations:** * Erciyes University, Kayseri, Turkey; † University of Maryland, Baltimore, Maryland, USA

**Keywords:** *Rickettsia akari*, rickettsialpox, vesicular rash, mites, diagnostic methods

**To the Editor:** Rickettsialpox is often described as a chickenpox-like disease and is caused by *Rickettsia akari*, a spotted fever group *Rickettsia* that is transmitted to humans by the bite of mites (*Liponyssoides sanguineus*). Although the mite host (typically a mouse) is widely distributed in cities, the disease is infrequently diagnosed. It is typically characterized in patients by the appearance of a primary eschar at the site of a mite bite followed by fever, headache, and development of a papulovesicular rash. Symptoms normally appear 9–14 days after the mite bite and are often unnoticed by the affected person. In documented rickettsialpox cases, the presence of a papule that ulcerates and becomes a scar approximately 0.5–3.0 cm in diameter is reported (*1*–*3*). Three to 7 days later, symptoms are more pronounced, with patients experiencing the sudden onset of chills, fever, and headache followed by myalgia and the appearence of generalized vesicular skin rashes. Less frequently, photophobia, conjunctival injection, cough, generalized lymphadenopathy, and vomiting are reported.

The first well-described clinical case of rickettsialpox was documented in New York City in 1946 (*1*). Historically, most documented rickettsialpox cases have occurred in large metropolitan areas of the United States (*2*), where the causative agent, *R. akari*, circulates primarily between the house mouse (*Mus musculus*) and its mite (*Liponyssoides sanguineus*). Recently, rickettsialpox cases have been reported from Croatia, Ukraine, South Africa, Korea, and North Carolina (*3*,*4*). *R. akari* was isolated from the blood of a patient suspected of having Mediterranean spotted fever rather than rickettsialpox; this was the first human isolate of *R. akari* reported in >40 years (*4*). Recent reports of a rickettsialpox case in North Carolina (*3*), *R. akari* seropositivity found in HIV-positive intravenous drug users in the inner city of Baltimore, Maryland (*5*), and in Central and East Harlem, New York City (*6*), as well as rickettsialpox cutaneous eruption in an HIV patient in New York (*7*), indicate that *R. akari* rickettsiosis is more common than previously thought and presents the risk of sporadic outbreaks worldwide.

We describe the clinical presentation of rickettsialpox in a 9-year-old boy from Nevpehir, located in the middle region of Turkey. Previously, a report from the Antalya area of Turkey described the prevalence of serum immunoglobulin (Ig) G antibodies in humans directed against *R. conorii* (spotted fever group *Rickettsia*) (*8*); however, rickettsialpox was not reported in Turkey. This report of what we believe to be the first described rickettsialpox case from Turkey further extends the recognized geographic distribution of *R. akari*.

A 9-year-old boy was admitted to the Kayseri hospital with fever >39°C and generalized papulovesicular exanthema. One week before admission, fever, profuse sweating, headache, and dysuria were present. On admission, physical examination indicated generalized vesicular, bullouse, and papular exanthema involving the lips and oral cavity. Notable pathologic findings at admission included a black eschar on the boy’s penis, bilateral prominent conjunctival ejection, and bilateral lower pulmonary rales. The leukocyte count was 13,300/mm^3^, hemoglobin was 14.49 mg/dL, and the platelet count was 544,000/mm^3^. Serum electrolytes and blood urea nitrogen levels and results of coagulation study and urine analysis were normal. Routine blood cultures taken 24 hours postadmission were sterile. Specific antibodies (IgG; IgM) against *Varicella* were not detected in serum samples (Duzen Laboratories, Ankara, Turkey). Additionally, the patient reported mice on the family’s farm.

A diagnosis of rickettsialpox was made and doxycycline treatment (200 mg/kg) was initiated. The patient serum sample was tested by indirect immunofluorescence assay (IFA) for IgG and IgM antibodies reactive with *R. akari* (Kaplan strain), *R. typhi* (Wilmington), *R. rickettsii* (Sheila Smith), and *R. conorii* (Malish 7). Serum IgG titers of 1/1280 and IgM of 1/40 to *R. akari* were detected and confirmed through cross-adsorption with rickettsial antigens (*R. rickettsii*, *R. conorii*) (*9*,*10*). Higher reciprocal titers were obtained against *R. akari* antigens than against *R. rickettsii* and *R. conorii* antigens (reciprocol titers of 1,024 vs. 512 and 512, respectively). We observed a difference in reduction in antibody titers against *R. akari* after adsorption with *R. akari* (Kaplan) (<16), *R. rickettsii* (256), and *R. conorii* (256). Antibodies against *R. typhi* were not detected. The IFA result confirmed the clinical diagnosis of *R. akari* infection. After 2 days of doxycycline treatment, the patient was afebrile, and the rickettsialpox infection resolved without scars or complications.

In summary, we present a case in which the presence of an eschar on the patient’s penis, the failure of lesions to appear in crops, the sparsity of lesions, and mice on the family’s farm led to a diagnosis of rickettsialpox, which was confirmed by cross-adsorption serologic findings. This case indicates that rickettsialpox is an emerging infectious disease in Turkey. We recommend further studies to define the prevalence of *R. akari* and the worldwide distribution of rickettsialpox.
